# P-1681. Accuracy of Xpert MTB/Rif Ultra for the Diagnosis of Tuberculosis in Indian Children

**DOI:** 10.1093/ofid/ofaf695.1855

**Published:** 2026-01-11

**Authors:** Dhruv Gandhi, Meenakshi Dey, Sonal Patil, Dhruv Mamtora, Ira Shah

**Affiliations:** Bai Jerbai Wadia Hospital for Children, Mumbai, India, West Monroe, LA; Bai Jerbai Wadia Hospital for Children, Mumbai, India, West Monroe, LA; Bai Jerbai Wadia Hospital for Children, Mumbai, India, West Monroe, LA; Bai Jerbai Wadia Hospital for Children, Mumbai, India, West Monroe, LA; Bai Jerbai Wadia Hospital for Children, Mumbai, India, West Monroe, LA

## Abstract

**Background:**

The diagnosis of pediatric tuberculosis (TB) remains challenging due to difficulties in specimen collection and the paucibacillary nature of the disease. While Mycobacteria Growth Indicator Tube (MGIT) culture is the gold standard, its long turnaround time limits clinical utility. Xpert MTB/Rif Ultra offers rapid and convenient detection, but its performance compared to MGIT in children requires further evaluation.The aim of this study is to assess the diagnostic performance of Xpert Ultra versus MGIT culture for detecting Mycobacterium tuberculosis (MTB) in Indian children with pulmonary or extrapulmonary TB.

**Table 1:**
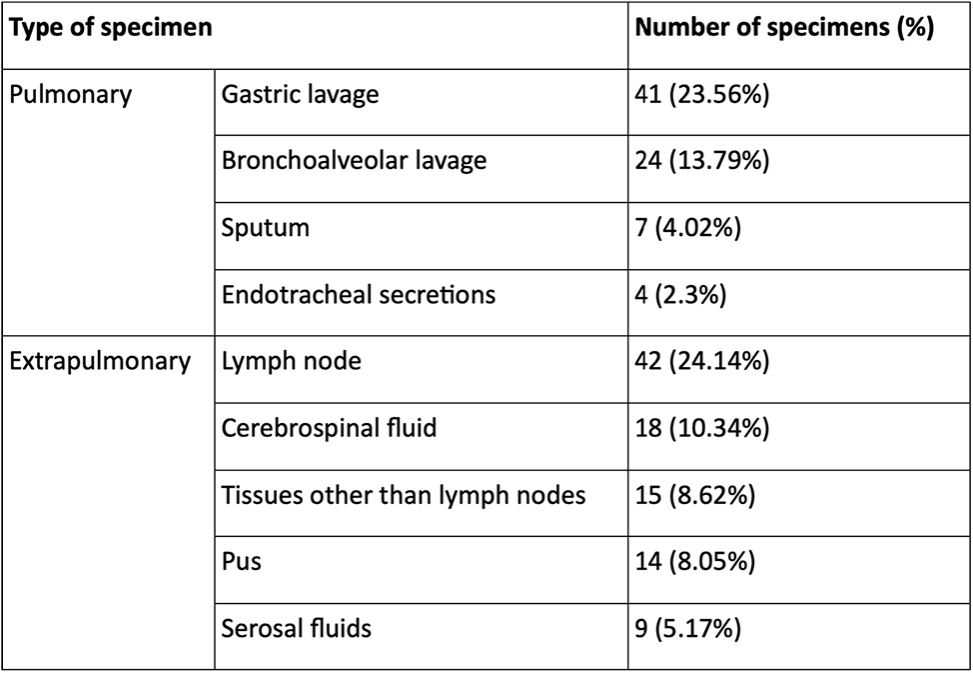
Type of specimens tested

**Table 2:**
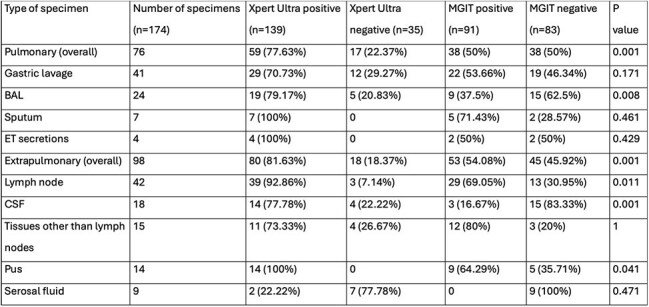
Results of Xpert Ultra and MGIT testing for each specimen type Note: MGIT- Mycobacteria Growth Indicator Tube, BAL- Bronchoalveolar lavage, ET- Endotracheal, CSF- Cerebrospinal fluid.

**Methods:**

A retrospective study was conducted between January 2021 and January 2023. All children < 18 years with clinically/microbiologically confirmed TB who underwent both Xpert Ultra and MGIT testing were included. Specimens included pulmonary (sputum, gastric lavage, bronchoalveolar lavage, endotracheal secretions) and extrapulmonary samples (lymph nodes, cerebrospinal fluid, pus, tissues, serosal fluid). Sensitivity, specificity, and kappa agreement between Xpert Ultra and MGIT were analyzed.

**Table 3:**
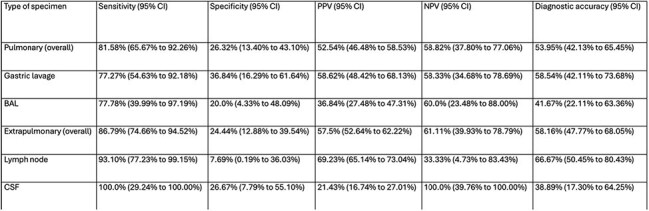
Sensitivity, specificity, PPV, NPV, and diagnostic accuracy for Xpert Ultra in comparison to MGIT culture for the detection of MTB for each specimen type Note: PPV- Positive predictive value, NPV- Negative predictive value, MGIT- Mycobacteria Growth Indicator Tube, MTB- Mycobacterium tuberculosis, CI- Confidence interval, BAL- Bronchoalveolar lavage, CSF- Cerebrospinal fluid.

**Table 4:**
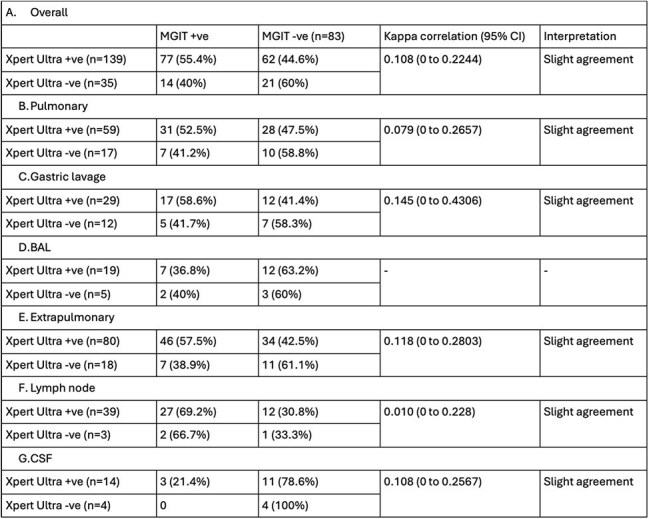
Kappa correlation analysis for Xpert Ultra and MGIT results for the detection of MTB for each specimen type Note: MGIT- Mycobacterial growth indicator tube, MTB- Mycobacterium tuberculosis, +ve- positive, -ve- negative, CI- Confidence interval, BAL- Bronchoalveolar lavage, CSF- Cerebrospinal fluid.

**Results:**

Among 174 specimens (76 pulmonary, 98 extrapulmonary), Xpert Ultra detected MTB in 79.89% (139/174) compared to MGIT in 52.3% (91/174). Overall sensitivity and specificity of Xpert Ultra versus MGIT culture for MTB detection was 84.62% and 25.3%, respectively. Sensitivity was higher for extrapulmonary (86.79%) than pulmonary samples (81.58%), but specificity was low overall (7.69% to 36.84%). Xpert Ultra missed 40% of MGIT-positive cases, while MGIT missed 44.6% of Xpert Ultra-positive cases. Kappa correlation was slight overall and for each specimen type.

**Conclusion:**

Xpert Ultra in comparison to MGIT showed higher MTB detection rates and higher sensitivity but lower specificity. Neither test alone was sufficient for microbiological confirmation. Combined Xpert Ultra and MGIT testing may improve pediatric TB diagnostic rates, particularly in high-burden settings such as India.

**Disclosures:**

All Authors: No reported disclosures

